# Evaluation of the Correlation of Reading Ability and Fixation Status in Patients With Neovascular Age-Related Macular Degeneration

**DOI:** 10.7759/cureus.91705

**Published:** 2025-09-06

**Authors:** Ryosuke Miki, Tomoaki Shiba, Toshiyuki Oshitari, Tomohiko Usui

**Affiliations:** 1 Ophthalmology, International University of Health and Welfare Narita Hospital, Chiba, JPN

**Keywords:** critical print size, maximum reading speed, minnesota low vision reading, neovascular age-related macular degeneration, reading visual acuity

## Abstract

Purpose

The purpose of this study was to evaluate the correlation of the reading ability obtained by Minnesota low vision reading (MNREAD) and the fixation status by microperimetry and morphological status in Japanese patients with neovascular age-related macular degeneration (nAMD).

Material and methods

We analyzed 25 consecutive nAMD patients and selected patients if the distance log MAR best corrected visual acuity (BCVA) of the studied eye was from 0.3 to 1.0. For reading ability, the reading visual acuity, maximum reading speed (MRS), and critical print size (CPS) were evaluated. Fixation stability (FS) was defined by the percentage of fixation points located within a circle of diameter 2° centered on all fixation points. For morphological status, central retinal thickness (CRT) and lesion size were evaluated. Multiple regression analyses were used to determine independent contributing factors for MRS and CPS. Single regression analyses were also used to determine the relationship between 2°FS, preferred retinal locus (PRL)-fovea distance, and other parameters.

Results

Fifteen patients had unilateral nAMD, and 10 patients had bilateral nAMD. The lesion size was identified as a factor independently contributing to the MRS. The BCVA of the fellow eye and 2°FS were identified as factors independently contributing to the CPS. The CRT and PRL-fovea distance were significantly negatively correlated with the 2°FS, and age was significantly negatively correlated with the PRL-fovea distance.

Conclusion

It is possible that there is a relationship between morphology, fixation status, and reading ability in Japanese patients with nAMD.

## Introduction

Age-related macular degeneration (AMD) is a major cause of blindness and severe visual loss in older people globally [1]. Intra-vitreous anti-vascular endothelial growth factor (VEGF) is standard therapy for neovascular age-related macular degeneration (nAMD). The results of a long-period follow-up study clarified that among nAMD patients, a significant portion were at risk for ongoing vision loss many years after treatment initiation [2]. Recently, an update of the Global Burden of Disease study reported that the estimated crude prevalence of AMD-related blindness decreased globally by almost 20%, while the prevalence of patients with moderate and severe vision impairment increased by 10% [3]. Central visual impairment leads to decreasing visual acuity and fixation stability (FS). If the other retinal area is used instead of the damaged fovea, it is called the preferred retinal locus (PRL) [4]. The FS decreased when used outside of the central retina [5]. The FS was also reported to be associated with visual acuity, morphological status, and PRL [6-9]. In addition, several studies confirmed the correlation between FS and reading ability [10-12]. However, the relationship between reading ability, status of PRL and FS, and morphological status in patients with nAMD is not fully understood, for example, the effect on the fellow eye's visual acuity. In addition, there are still limited reports on research targeting Japanese patients.

The purpose of the present study was thus to evaluate the correlation with reading ability obtained by Minnesota low vision reading (MNREAD), FS, and the PRL-fovea distance, calculated by microperimetry and morphological status, in Japanese patients with nAMD.

## Materials and methods

This study was cross-sectional and was approved by the Institutional Review Board of the International University of Health and Welfare Narita Hospital (22-Nr-001). All patients provided their written informed consent for participation in the study in accordance with the tenets of the Declaration of Helsinki.

We analyzed 25 consecutive Japanese nAMD patients who visited the Department of Ophthalmology of International University of Health and Welfare Narita Hospital in the years from May 1, 2022, to November 1, 2023. Patients were selected if the distance log MAR best corrected visual acuity (BCVA) of the study eye was from 0.3 to 1.0, and if both eyes had nAMD, the eye with better BCVA was studied because reading ability is higher in the better eye. Eleven patients were before intravitreal anti-VEGF therapy, and 14 subjects were already administered intravitreal anti-VEGF therapy when the evaluation was conducted.

Access to reading ability

Reading ability was assessed by MNREAD [13]. We used the Japanese version, which is named MNREAD-J, in this study [14,15]. The MNREAD was also developed for tablets, and we thus used the MNREAD-J iPad version (MNREAD iPad App, version 1.17). A detailed explanation of the methods used to measure the MNREAD-J has been reported [14,15]. The test was conducted under a corrected status after enough practice trials were provided.

Briefly, the sentences of the MNREAD-J involve a meaning, and each sentence is set vertically and comprises 30 characters (10 characters/line). The sentences can be chosen in decreasing print size from 1.3 to 0.5 log MAR. The patients read the sentences under monocular conditions with occluding glasses because some study patients mentioned no abnormalities in the fellow eye. The reading charts were located initially at a distance of 30 cm.

The tablet was held horizontally, the overhead light was kept on, with no additional lighting, and the tablet screen brightness was set at 200 cd/m2, because this light level is reported to be not strong enough to disturb low-vision patients [16]. The following reading ability parameters were assessed: 1. reading visual acuity (RA, log MAR), the smallest print size that the person can read without errors; 2. maximum reading speed (MRS) in words per minute (wpm), a patient’s reading speed when reading is not limited by print size; 3. critical print size (CPS, log MR), the smallest print size that the patient can read at maximum speed [17].

Evaluation of fixation status

The FS of the target eye was measured using a microperimeter (MP-3, Nidek, Aichi, Japan) under dark room conditions. Measurements were performed for 30 seconds without pupil dilation and the fellow eye occluded, with a background luminance of 31.4 asb, and a fixation light of a 0.2 degrees’ thickness by 1 degree of red cross [18]. The microperimeter recorded eye movements at a sampling rate of 30 Hz. During the measurement, the MP-3 uses auto-tracking and auto-alignment technologies to enhance measurement accuracy [19]. The FS was defined by the percentage of fixation points located within a circle of diameter 2°, centered on all fixation points [20]. In addition, we assessed the PRL-fovea distance, which was defined as the degree of eccentric fixation point from the fovea [10].

Measurements of other ocular parameters

The estimated disease duration of nAMD (months) and the distance of best corrected visual acuity (log MAR) in both eyes were evaluated. Optic coherence tomography (OCT) imaging was conducted with a DRI Triton Swept Source OCT system (Topcon, Tokyo, Japan) to evaluate the target eyes' morphological status, and the following parameters were measured: central retinal thickness (CRT; μm) and lesion size (mm2). Lesion size was calculated manually from the fundus image obtained by OCT. All OCT parameters were calculated by author RM masking the information.

Diagnosis of nAMD

nAMD was diagnosed primarily based on the findings of ophthalmoscopy, fundus photography, and OCT angiography images according to the Japanese clinical guidelines for nAMD [21].

Statistical analyses

Data are presented as mean ± standard deviation for continuous variables. Single and multiple regression analyses were used to determine independent contributing factors for MRS and CPS. Single regression analyses were also used to determine the relationship between 2°FS, PRL-fovea distance, and other parameters. All statistical analyses were performed by EZR (Saitama Medical Center, Jichi Medical University, Saitama, Japan, ver. 1.54) [22]. Probability (p)-values <0.05 were considered significant.

## Results

Table 1 shows the characteristics of 15 (60%) men and 10 (40%) women included in the study. The mean patient age was 75.4± 7.9 (mean±SD) years. Fifteen (60%) patients had unilateral nAMD, and 10 (40%) patients had bilateral nAMD. The duration of the disease period of nAMD was 33.0 ± 39.9 (mean±SD) months.

**Table 1:** Characteristics of all patients (N=25) BCVA; best corrected visual acuity, nAMD; neovascular age-related macular degeneration, CRT; central retinal thickness, PRL; preferred retinal locus, FS; fixation stability, RA: reading acuity, MRS; maximum reading speed, wpm; words per minute, CPS; clinical print size

Variables (N=25)	Mean ±SD
Age (years)	75.4 ± 7.9
Male: Female	15 (60%): 10 (40%)
Unilateral: Bilateral	15(60%): 10 (40%)
BCVA (study eye, log MAR)	0.62 ± 0.24
BCVA(fellow eye, log MAR)	0.41 ± 0.92
Duration of nAMD (months)	33.0 ± 39.9
CRT (μm)	260.6 ± 104.3
Lesion size (mm^2^)	5.9 ± 4.6
PRL-fovea distance (°)	1.62 ± 2.47
2°FS (%)	86.48 ± 21.35
RA (study eye, log MAR)	0.62 ± 0.22
MRS (wpm)	174.8 ± 80.9
CPS (log MAR)	0.82 ± 0.21

Table 2 shows the results of the single and multiple regression analyses assessing the independent contributions of MRS. Because the correlation coefficients were >0.9 among BCVA (study eye) and RA, we selected BCVA (study eye) as the explanatory variable that showed stronger correlations with the objective variables. The lesion size was identified as a factor independently contributing to the MRS (partial regression coefficient, B = -6.49, t value = -2.11, and p value = 0.04).

**Table 2:** Results of single and multiple regression analyses of factors independently contributing to the maximum reading speed (N=25) r; correlation coefficient, B; partial regression coefficient, BCVA; best corrected visual acuity, nAMD; neovascular age-related macular degeneration, CRT; central retinal thickness, PRL; preferred retinal locus, FS; fixation stability, RA; reading acuity, CPS; clinical print size

	Single regression analysis (N=25)		Multiple regression analysis (N=25)		
Variables	r	p	B	t	p
Age (years)	0.19	0.37			
Male: Female	-0.11	0.59			
Unilateral: Bilateral	-0.01	0.96			
BCVA (study eye, log MAR)	-0.53	<0.01	-93.74	-1.58	0.13
BCVA(fellow eye, log MAR)	-0.08	0.71			
Duration of nAMD (months)	-0.34	0.09	-0.63	-1.89	0.07
CRT (μm)	0.06	0.77			
Lesion size (mm^2^)	-0.46	0.02	-6.49	-2.11	0.04
PRL-fovea distance (°)	-0.34	0.10			
2°FS (%)	0.44	0.03	0.83	1.32	0.20
RA (study eye, log MAR)	-0.45	0.02			
CPS (log MAR)	-0.22	0.29			

The results of single and multiple regression analyses of factors independently contributing to the CPS are shown in Table 3. We also selected BCVA (study eye) as the explanatory variable that showed stronger correlations than RA. The BCVA of the fellow eye (B = -0.11, t value = -2.95, p value = 0.008) and 2°FS (B = -3.35×103, t value = 2.14, p value = 0.04) were identified as factors independently contributing to the CPS.

**Table 3:** Results of single and multiple regression analyses of factors independently contributing to the clinical print size (N=25) r; correlation coefficient, B; partial regression coefficient, BCVA; best corrected visual acuity, nAMD; neovascular age-related macular degeneration, CRT; central retinal thickness, PRL; preferred retinal locus, FS; fixation stability, RA; reading acuity, MRS; maximum reading speed; wpm; words per minute

	Single regression analysis (N=25)		Multiple regression analysis (N=25)		
Variables	r	p	B	t	p
Age (years)	-0.17	0.41			
Male: Female	-0.23	0.26			
Unilateral: Bilateral	-0.08	0.71			
BCVA (study eye, log MAR)	0.48	0.01	0.29	1.90	0.07
BCVA(fellow eye, log MAR)	-0.57	<0.01	-0.11	-2.95	<0.01
Duration of nAMD (months)	0.02	0.92			
CRT (μm)	0.31	0.13			
Lesion size (mm^2^)	0.06	0.76			
PRL-fovea distance (°)	0.29	0.16			
2°FS (%)	-0.50	0.01	-3.35×10^3^	-2.14	0.04
RA (study eye, log MAR)	0.43	0.03			
MRS (wpm)	-0.22	0.29			

Lastly, we conducted single regression analyses to determine correlation coefficients (r) among 2°FS, PRL-fovea distance, and all parameters (Table 4). The CRT (r= -0.42, p value = 0.04) and PRL-fovea distance (r= -0.76, p value=<0.0001) were significantly negatively correlated with the 2°FS. Age (r= -0.43, p value = 0.03) and 2°FS (r= -0.76, p value=<0.0001) were significantly negatively correlated with the PRL-fovea distance. We also examined the direction of eccentric fixation, but found no correlation between reading ability (CPS, r = -0.06, p value = 0.78, MRS, r = -0.04, p value = 0.83).

**Table 4:** Correlation coefficients of single regression analyses between fixation stability, preferred retinal locus-fovea distance, and all parameters (N=25) r; correlation coefficient, BCVA; best corrected visual acuity, nAMD; neovascular age-related macular degeneration, CRT; central retinal thickness, PRL; preferred retinal locus, FS; fixation stability, RA; reading acuity

	2°FS (N=25)		PRL-fovea distance (N=25)	
Variables	r	p	r	p
Age (years)	0.36	0.08	-0.43	0.03
Male: Female	-0.01	0.97	0.04	0.85
Unilateral: Bilateral	-0.02	0.93	0.10	0.64
BCVA (study eye, log MAR)	-0.28	0.18	0.16	0.46
BCVA(fellow eye, log MAR)	0.16	0.47	-0.11	0.60
Duration of nAMD (months)	-0.22	0.28	0.26	0.21
CRT (μm)	-0.42	0.04	0.35	0.09
Lesion size (mm^2^)	-0.20	0.33	0.30	0.14
PRL-fovea distance (°)	-0.76	<0.001		
2°FS (%)			-0.76	<0.001
RA (study eye, log MAR)	-0.22	0.28	0.11	0.59

## Discussion

The current study evaluated factors contributing to reading ability in the target eye in nAMD cases with BCVA from 0.3 to 0.5 from the point of view of fixation status and morphological condition. Ten cases of 25 included bilateral nAMD, and BCVA in the fellow eyes varied from light perception to 1.0. All analyses of target eyes were conducted according to these patients' backgrounds. Our multiple regression analysis revealed that lesion size was identified as an independent contributing factor for MRS, while the status of the fellow eye did not contribute to MRS in the study eye. A previous study of advanced AMD has shown that reading ability and geographic atrophy size are contributing factors to the quality of vision measured by the 25-Item National Eye Institute Visual Function Questionnaire [23]. A study on Japanese patients with bilateral nAMD also reported that the MRS contributes greatly to social dependency [24]. In this study, 10 out of 25 consecutive cases (40%) were bilateral nAMD. We previously analyzed a total of 259 Japanese patients with nAMD and reported that the rate of cases with bilateral development was 13% [25]. Thus, the possibility of nAMD developing in both eyes in the future is not low. Considering reading ability, even in cases of unilateral nAMD, it is meaningful to suppress the progression of lesion size over the long term to maintain social independence such as maintaining reading speed.

Our multiple regression analysis revealed that BCVA of the fellow eye and 2°FS were selected as independent contributing factors of CPS. In single regression analysis, CPS showed a positive correlation with the RA and BCVA of the study eye. This result means that the better the visual acuity of the target eye, the smaller the print size at which reading is possible. On the other hand, a negative correlation was found between BCVA of the fellow eye and CPS. This result indicated that the worse the visual acuity of the fellow eye, the smaller the print size at which reading is possible. Our multiple regression analysis suggested that the BCVA of the fellow eye contributes more strongly than the status of visual acuity in the study eye for CPS. This result suggests that the ability of CPS is improved when vision in both eyes is affected.

Finally, we conducted a single regression analysis to determine factors related to FS and PRL-fovea distance. The PRL-fovea distance and CRT were significantly negatively correlated with FS. It has been reported that FS is closely related to PRL and reading ability, and our results reconfirmed this in the study [6-9]. Our results also showed that 2°FS was an important factor in CPS, and CRT was related to factors related to 2°FS. From the above, it was suggested that maintaining optimal CRT through continuous treatment for nAMD was very important in maintaining reading ability. BCVA of the fellow eye did not correlate with 2°FS and the PRL-fovea distance, and age showed a negative correlation with the PRL-fovea distance. This suggested that eccentric fixation might be easier to obtain in younger patients, but unfortunately, the PRL-fovea distance did not show a correlation with reading ability in this study. We also examined the direction of eccentric fixation, but found no correlation with reading ability. In this study, the mean value of the PRL-fovea distance was 1.62°, and it is smaller than previous reports of patients who needed low vision rehabilitation [10]. However, the exact mechanisms behind PRL development are still elusive. The PRL usually develops within six months of vision loss [5]. A re-examination will thus be needed among bilateral nAMD patients with a longer follow-up period.

Recently, intravitreal anti-VEGF therapy for nAMD has been shifting from as-needed treatment to continuous proactive treatment. This is a large financial burden on patients, especially in Japan. A previous study has reported that intravitreal anti-VEGF therapy significantly improves reading ability [26]. The results of this study may contribute to maintaining the motivation of both therapists and nAMD patients regarding the currently recommended, proactive intravitreal anti-VEGF therapy.

This study has some important limitations. First, our nAMD patients included both cases before and after treatment intervention. It is a possibility that this may have influenced CRT. The activity of choroidal neovascularization may have slightly influenced the results of this study, and prospective reevaluation is needed in the future, including the presence or absence of activity before and after treatment. Second, there are various types of nAMD, but currently, this study did not examine them by disease type due to the small number of our patients. Third, all evaluations were conducted with the same brightness of illumination. It has been reported that reading ability improved with increasing illumination [15]. Further investigation under different brightness levels will thus be needed. Fourth, our study had only 25 subjects. Due to the small number of subjects, our statistics and the conclusions we could draw from them might include some limitations. Finally, we did not evaluate the comorbidities, especially cognitive status. There was a possibility that these factors might have affected the reading ability results. Clearly, further prospective studies with a larger number of patients with more varied fellow eye status will be needed to better understand reading ability in nAMD patients.

## Conclusions

This study evaluated the correlation between reading ability obtained by MNREAD and fixation status in Japanese patients with nAMD. The lesion size was identified as a factor independently contributing to MRS. The BCVA of the fellow eye and 2°FS were identified as factors independently contributing to the CPS. The CRT and the PRL-fovea distance were significantly negatively correlated with 2°FS, and age was significantly negatively correlated with the PRL-fovea distance. It is possible that there is a relationship between morphological fixation status and reading ability in Japanese patients with nAMD.
